# Associations of circulating GDF15 with combined cognitive frailty and depression in older adults of the MARK-AGE study

**DOI:** 10.1007/s11357-023-00902-6

**Published:** 2023-09-16

**Authors:** Bastian Kochlik, Catrin Herpich, María Moreno-Villanueva, Susanne Klaus, Ursula Müller-Werdan, Birgit Weinberger, Simone Fiegl, Olivier Toussaint, Florence Debacq-Chainiaux, Christiane Schön, Jürgen Bernhard, Nicolle Breusing, Efstathios S. Gonos, Claudio Franceschi, Miriam Capri, Ewa Sikora, Antti Hervonen, Mikko Hurme, P. Eline Slagboom, Martijn E. T. Dollé, Eugene Jansen, Tilman Grune, Alexander Bürkle, Kristina Norman

**Affiliations:** 1 Department of Nutrition and Gerontology, German Institute of Human Nutrition Potsdam-Rehbruecke (DIfE), Nuthetal, Germany; 2 Department of Molecular Toxicology, German Institute of Human Nutrition Potsdam-Rehbruecke (DIfE), Nuthetal, Germany; 3 Food4Future (F4F), c/o Leibniz Institute of Vegetable and Ornamental Crops (IGZ), Theodor-Echtermeyer-Weg 1, 14979 Grossbeeren, Germany; 4 Institute of Nutritional Science, University of Potsdam , Potsdam, Germany; 5 Department of Geriatrics and Medical Gerontology, Charité – Universitätsmedizin Berlin, corporate member of Freie Universität Berlin and Humboldt-Universität Zu Berlin, Berlin, Germany; 6 Molecular Toxicology Group, Department of Biology, University of Konstanz, Constance, Germany; 7 Human Performance Research Centre, Department of Sport Science, University of Konstanz, Constance, Germany; 8 Department of Physiology of Energy Metabolism, German Institute of Human Nutrition Potsdam-Rehbruecke (DIfE), Nuthetal, Germany; 9 Protestant Geriatric Center Berlin (EGZB), Berlin, Germany; 10 Research Institute for Biomedical Aging Research, Universität Innsbruck, Rennweg 10, 6020 Innsbruck, Austria; 11 UMIT TIROL, Eduard-Wallnöfer-Zentrum 1, 6060 Hall in Tirol, Austria; 12 URBC-Narilis, University of Namur, Rue de Bruxelles 61, B-5000 Namur, Belgium; 13 BioTeSys GmbH, Schelztorstraße 54-56, 73728 Esslingen, Germany; 14 Institute of Nutritional Medicine, Department of Applied Nutritional Science/Dietetics, University of Hohenheim, Stuttgart, Germany; 15 Institute of Biological Research and Biotechnology, National Hellenic Research Foundation (NHRF, 48 Vas. Constantinou Ave, 11635 Athens, Greece; 16 Institute of Information Technology, Mathematics and Mechanics, Department of Applied Mathematics, National Research Lobachevsky State University of Nizhny Novgorod, Nizhny Novgorod, Russia; 17 Department of Medical and Surgical Sciences, University of Bologna-Alma Mater Studiorum, Bologna, Italy; 18 Alma Mater Research Institute On Global Challenges and Climate Change (Alma Climate), University of Bologna, Bologna, Italy; 19 Laboratory of the Molecular Bases of Ageing, Polish Academy of Sciences, Nencki Institute of Experimental Biology, Warsaw, Poland; 20 Faculty of Medicine and Health Technology, Tampere University, Tampere, Finland; 21 Department of Molecular Epidemiology, Leiden University Medical Center, Leiden, The Netherlands; 22 Centre for Health Protection, National Institute for Public Health and the Environment (RIVM), P.O. Box 1, 3720 BA Bilthoven, The Netherlands; 23 Faculty of Chemistry, Department of Physiological Chemistry, University of Vienna, Vienna, Austria; 24 German Centre for Cardiovascular Research (DZHK), Partner Site Berlin, Berlin, Germany

**Keywords:** GDF15, Cognitive frailty, Depression, Aging, Biomarker

## Abstract

Growth differentiation factor-15 (GDF15) might be involved in the development of cognitive frailty and depression. Therefore, we evaluated cross-sectional associations of plasma GDF15 with combined cognitive-frailty-and-depression in older (i.e. ≥ 55 years) and younger adults of the MARK-AGE study. In the present work, samples and data of MARK-AGE (“European study to establish bioMARKers of human AGEing“) participants (*N* = 2736) were analyzed. Cognitive frailty was determined by the global cognitive functioning score (GCF) and depression by the Self-Rating Depression Scale (SDS score). Adults were classified into three groups: (I) neither-cognitive-frailty-nor-depression, (II) either-cognitive-frailty-or-depression or (III) both-cognitive-frailty-and-depression. Cross-sectional associations were determined by unadjusted and by age, BMI, sex, comorbidities and hsCRP-adjusted linear and logistic regression analyses. Cognitive frailty, depression, age and GDF15 were significantly related within the whole study sample. High GDF15 levels were significantly associated with both-cognitive-frailty-and-depression (adjusted β = 0.177 [0.044 – 0.310], *p* = 0.009), and with low GCF scores and high SDS scores. High GDF15 concentrations and quartiles were significantly associated with higher odds to have both-cognitive-frailty-and-depression (adjusted odds ratio = 2.353 [1.267 – 4.372], *p* = 0.007; and adjusted odds ratio = 1.414 [1.025 – 1.951], *p* = 0.035, respectively) independent of age, BMI, sex, comorbidities and hsCRP. These associations remained significant when evaluating older adults. We conclude that plasma GDF15 concentrations are significantly associated with combined cognitive-frailty-and-depression status and, with cognitive frailty and depressive symptoms separately in old as well as young community-dwelling adults.

**Supplementary Information:**

The online version contains supplementary material available at 10.1007/s11357-023-00902-6.

## Introduction

Growth differentiation factor-15 (GDF15), a member of the TGF-β superfamily, is a signal molecule induced by different age-related stressors [1] such as inflammation [2]. GDF15 levels are increased in older age and as GDF15 plays an important role in the aging process itself, it has been suggested to be a biomarker of aging [1, 3]. Additionally, GDF15 has been proposed to be part of the senescence-associated secretory phenotype (SASP) that represents the pro-inflammatory secretome released by senescent cells [1]. Intriguingly, GDF15 can exert or mediate both anti-inflammatory and pro-inflammatory effects, which seem to be age- as well as concentration-dependent [1]. High circulating GDF15 concentrations have been linked to both age-related conditions, such as cardiovascular diseases [4] and frailty [5], as well as to all-cause mortality [6]. Furthermore, high GDF15 in older age was associated with cognitive impairment [7, 8], as well as with lower cognitive function [9].

The frailty syndrome can be characterized by an accumulation of physical, cognitive or psychological function deficits that result in an enhanced vulnerability for negative health outcomes [10]. In contrast to the primarily studied physical frailty domain [11], the cognitive frailty domain, as a novel complementary concept to physical frailty [12], is less studied, and therefore little is known about contributing factors and underlying mechanisms. Depression, a heterogeneous mental health disorder that can also affect physical health, is one of the most important health problems worldwide [13] and a common chronic disease that can lead to impaired psychosocial functioning and to diminished quality of life [13, 14]. Furthermore, depressive symptoms (depression) and frailty are associated with each other, can coexist and share pathophysiological mechanisms, such as inflammation [15, 16]. Since increased inflammation and advanced age are associated with frailty [17, 18], with a decline in brain and cognitive function [14, 19] and with depression [14, 20], we hypothesized that GDF15 is associated with both cognitive frailty and depression.

Studies investigating the relation of circulating GDF15 with combined cognitive frailty and depression are lacking, so far, especially in studies including both older and younger adults. Therefore, we first evaluated whether circulating GDF15 concentrations (I) are altered in adults with both cognitive frailty and depressive symptoms, (II) are associated with a higher likelihood to be cognitive frail or to have depressive symptoms, and (III) are elevated in adults with either cognitive frailty or depressive symptoms. Furthermore, we determined if associations can be confirmed in analyses considering only older adults.

## Methods

### Study population and participant characteristics

For the present study we analyzed participants of the MARK-AGE study (“European study to establish bioMARKers of human AGEing“), which is a cross-sectional study comprising multiple European populations that aims to identify reliable biomarkers of human aging [21]. Participants either belonged to (I) randomly recruited age-stratified individuals from the general population covering the age range 35—74 years (RASIG), or (II) subjects born from a long-living parent belonging to a family with long living sibling(s) from the Genetics of Healthy Ageing project (GEHA), therefore referred as GEHA offspring (GO) together with spouses of GEHA offsprings (SGO) [21, 22]. Details on recruitment of participants, standardized determination of participant characteristics (e.g. age, sex, body mass index [BMI], number of comorbidities (reflecting participants health status)), participants cognitive frailty and depression status as well as immunological biomarker measures like high-sensitive C-reactive protein (hsCRP, [in mg/L]) have been described elsewhere [22, 23]. Ethical approval for the study was given by the local Research Ethics Committees of each recruitment center. All participants gave written informed consent to participate. The MARK-AGE study was conducted in accordance with the Declaration of Helsinki (1964). The study has retrospectively been registered at the German Clinical Trials Register (DRKS00007713).

### Cognitive frailty status and depression status

Cognitive frailty was determined by the global cognitive functioning (GCF) score, and adults were defined and classified as cognitively frail when scoring below the 10^th^ percentile on the GCF score [24, 25]. Adults scoring above the 10^th^ percentile on the GCF score were defined as cognitively robust. The GCF score was based on different cognitive functioning tests including the (I) 15-Picture Word Learning test to evaluate immediate and delayed memory function [26], (II) Stroop test to evaluate cognitive flexibility [27], and (III) Digit Symbol Substitution test to determine cognitive speed [28]. Scores of these cognitive tests were first transformed into z-scores, which were subsequently combined into the GCF score.

Depression status was determined by the Self-Rating Depression Scale (SDS) according to Zung [29], ranging from 20 to 80 points, which was subsequently transformed into the SDS Index (SDS score), ranging from 25 to 100 points. This is a validated questionnaire that can be used in various age groups to measure and screen depression status [29, 30], which was filled out by trained interviewer together with the study participants. Adults with an SDS score ≥ 50 points were considered to have depressive symptoms, and thus, defined and classified as “adults with depressive symptoms”. Adults with an SDS score < 50 points were defined and classified as “adults without depressive symptoms”.

Adults were then classified into three groups, depending on their combined cognitive-frailty-depression status, as follows: (I) neither-cognitive-frailty-nor-depression, (II) either-cognitive-frailty-or-depression, and (III) both-cognitive-frailty-and-depression.

### GDF15 measurement in plasma

Venous blood was collected by venipuncture in the morning after an overnight fast and processed within 3–5 h to obtain aliquots of whole blood, serum and plasma, which were immediately frozen and stored at − 80 °C. Plasma GDF15 concentrations [in pg/mL] were measured according to manufacturer’s instructions of the commercial human GDF-15/MIC-1 ELISA kit (BIOVENDOR, Brno, Czech Republic), with intra- and inter-assay coefficients of variability of 6.3 — 7.2% and 2.9 — 5.6%, respectively.

### Statistical analyses

Participant characteristics and plasma GDF15 concentrations as well as hsCRP concentrations are reported for the total study population, and separately according to their cognitive frailty and depression status. Continuous variables are shown as mean ± standard deviations (SD) or as median (interquartile range [IQR]). When necessary, GDF15 concentrations were logarithmically transformed (LnGDF15) and back-transformed values are shown by geometric means with 95% confidence intervals (95% CI). Categorical variables are shown as amount with frequencies (n [%]).

Differences in characteristics, plasma GDF15 and hsCRP concentrations, and both GCF and SDS scores between the three status groups were determined by χ2-test (for categorical variables) and by one-way ANOVA (for continuous variables). Bivariate correlations between plasma GDF15, age, BMI, both GCF and SDS scores and hsCRP concentrations were determined by Pearson correlation coefficient (r) or by Spearman rank correlation coefficient (ρ). Participants were categorized into nine age-groups (35 – 39 years, 40 – 44 years, 45 – 49 years, 50 – 54 years, 55 – 59 years, 60 – 64 years, 65 – 69 years, 70 – 74 years and plus  75 years) to evaluate the age-dependent prevalence of both-cognitive-frailty-and-depression as well as cognitive frailty and depression separately.

Associations between GDF15 concentrations and combined cognitive-frailty-depression status were determined by unadjusted (β coefficient [β]) and by age, BMI, sex, comorbidities and hsCRP-adjusted (adjusted β) general linear models. The likelihood, i.e. odds ratios (OR), to be in one of the three groups (cognitive-frailty-depression status as dependent variable) with higher GDF15 concentrations (either LnGDF15 units or GDF15 quartiles as predictors) was evaluated by unadjusted (OR; crude model) and by age, BMI, sex, comorbidities and hsCRP-adjusted (adjusted OR; model 1) multinomial logistic regression analyses. GDF15 quartiles were as follows: quartile (Q)1: GDF15 ≤ 573.24 pg/mL, Q2: GDF15 = 573.25 – 758.13 pg/mL, Q3: GDF15 = 758.14 – 1013.06 pg/mL and Q4: GDF15 ≥ 1013.07 pg/mL. Associations of both SDS and GCF scores with GDF15 concentrations were evaluated by unadjusted (β; crude model) and by age, BMI, sex, comorbidities and hsCRP-adjusted (adjusted β; model 1) linear regression models. The odds to have cognitive frailty or depressive symptoms with higher GDF15 concentrations (LnGDF15 or GDF15 quartiles) was determined by unadjusted (OR; crude model) and by age, BMI, sex, comorbidities and hsCRP-adjusted (adjusted OR; model 1) logistic regression models.

We then focused our analyses on older adults aged ≥ 55 years. This age cutoff was chosen since there was a constant increase in cognitive frailty, in depression and in both-cognitive-frailty-and-depression prevalence in the age-groups 55 years and older (Supplemental Figure S1). Furthermore, the age of 55 years is defined as cutoff age that discriminates younger and older adults within the RASIG cohort of the MARK-AGE study population.

All statistical analyses were carried out using IBM SPSS Statistics software (Version 25; IBM, Armonk, NY, USA). GraphPad Prism (Version 9; GraphPad Software Inc., Boston, MA, USA) and Microsoft PowerPoint (Microsoft Corporation, Redmond, WA, USA) was used for figure preparation. Statistically significant differences and associations were considered to be present at *P* < 0.05.

## Results

The present study included a total of 2736 adults aged 57.7 ± 10.9 years (age range: 35 – 81 years) and consisted of 52.0% women. Descriptive data of the total study population, and according to their cognitive-frailty-and-depression status are shown in Table 1. Prevalence of the three cognitive-frailty-and-depression groups are as follow: 80.2% neither-cognitive-frailty-nor-depression, 17.7% either-cognitive-frailty-or-depression and 2.1% both-cognitive-frailty-and-depression. Adults with both-cognitive-frailty-and-depression are significantly older, have significantly lower GCF scores and higher SDS scores, and have significantly higher plasma GDF15 than adults of the other two groups. Adults with both-cognitive-frailty-and-depression also have significant higher BMI and more comorbidities, whereas sex distribution and hsCRP concentrations were similar between groups (Table 1). The prevalence of both-cognitive-frailty-and-depression combined as well as of cognitive frailty and of depression, separately, increases with advancing age (Supplemental Figure S1). Significant correlations of age, GDF15, GCF and SDS indicate a relationship between both conditions, age and GDF15 (Supplemental Table S2). Additionally, there is a significant positive correlation between GDF15 and hsCRP.

**Table 1:** Participant characteristics and GDF15 concentrations according to cognitive-frailty-and-depression status of all adults (*n* = 2736) of the MARK-AGE study

	Total	no-cognitive-frailty-no-depression	either-cognitive-frailty-or-depression	both-cognitive-frailty-and-depression	*p*-value
N [%]	2736 (100)	2193 (80.2)	485 (17.7)	58 (2.1)	-
GCF score [points]	0.134 ± 2.777	0.576 ± 2.335 ^a^	-1.309 ± 3.625 ^b^	-4.527 ± 1.159 ^c^	** < 0.001**
SDS score [points]	38.2 ± 9.6	35.4 ± 7.1 ^a^	48.2 ± 10.6 ^b^	57.1 ± 6.7 ^c^	** < 0.001**
Women [n (%)]	1424 (52.0)	1136 (79.8)	258 (18.1)	30 (2.1)	0.856 ^#^
Men [n (%)]	1312 (48.0)	1057 (80.6)	227 (17.3)	28 (2.1)	
Age [years]	57.7 ± 10.9	57.0 ± 10.9 ^a^	60.5 ± 10.4 ^b^	64.1 ± 9.1 ^c^	** < 0.001**
BMI [kg/m^2^]	26.3 ± 4.4	26.2 ± 4.4 ^a^	26.7 ± 4.4 ^b^	27.8 ± 4.3 ^b^	**0.002**
Comorbidities [n]	1.0 (2.0)	1.0 (2.0)	1.0 (3.0)	2.0 (2.0)	** < 0.001**
hsCRP [mg/L] ^1^	1.27 (2.03)	1.25 (2.00)	1.36 (2.17)	1.24 (1.35)	0.313
GDF15 [pg/mL] ^2^	773.7 (760.7; 787.0)	750.0 (735.4; 764.8) ^a^	860.1 (826.9; 894.6) ^b^	1058.9 (940.6; 1192.2) ^c^	** < 0.001**
		764.3 (752.4; 776.4) ^a^	810.0 (783.4; 837.5) ^b^	911.4 (826.0; 1005.7) ^b^	< 0.001*

Data are shown as mean ± standard deviation or as median (interquartile range). ^1^ hsCRP: *N* = 2607 (*n* = 129 participants with hsCRP concentration = 0 mg/L were excluded). ^2^ Data for GDF15 concentrations are shown as geometric mean (95% confidence interval (95% CI)) of back-transformed LnGDF15 values. Differences between groups are determined by one-way ANOVA with Bonferroni post-hoc test or Kruskal–Wallis-test for continuous variables and by ^#^ Chi-square-test for categorical variables. * ANCOVA: adjusted for age, BMI and sex. Superscript letters indicate statistically significant differences between frailty groups. Significance considered at *p* < 0.05. BMI, body mass index; GCF, global cognitive functioning; GDF15, growth differentiation factor-15; hsCRP, high-sensitive C-reactive protein; SDS, self-rating depression scale

Descriptive data of the older adults (age ≥ 55 years; *n* = 1712; 51.5% women) are shown in Supplemental Table S1. Here, older adults with both-cognitive-frailty-and-depression have significantly higher GDF15 than the other two groups. Furthermore, older adults show similar results like the whole study population regarding GCF scores, SDS scores, age, comorbidities, hsCRP and sex distribution (Supplemental Table S1).

### Combined cognitive-frailty-and-depression is age, BMI, sex, comorbidities and hsCRP-independently associated with high plasma GDF15 concentrations in adults of the MARK-AGE study

**Fig. 1:**
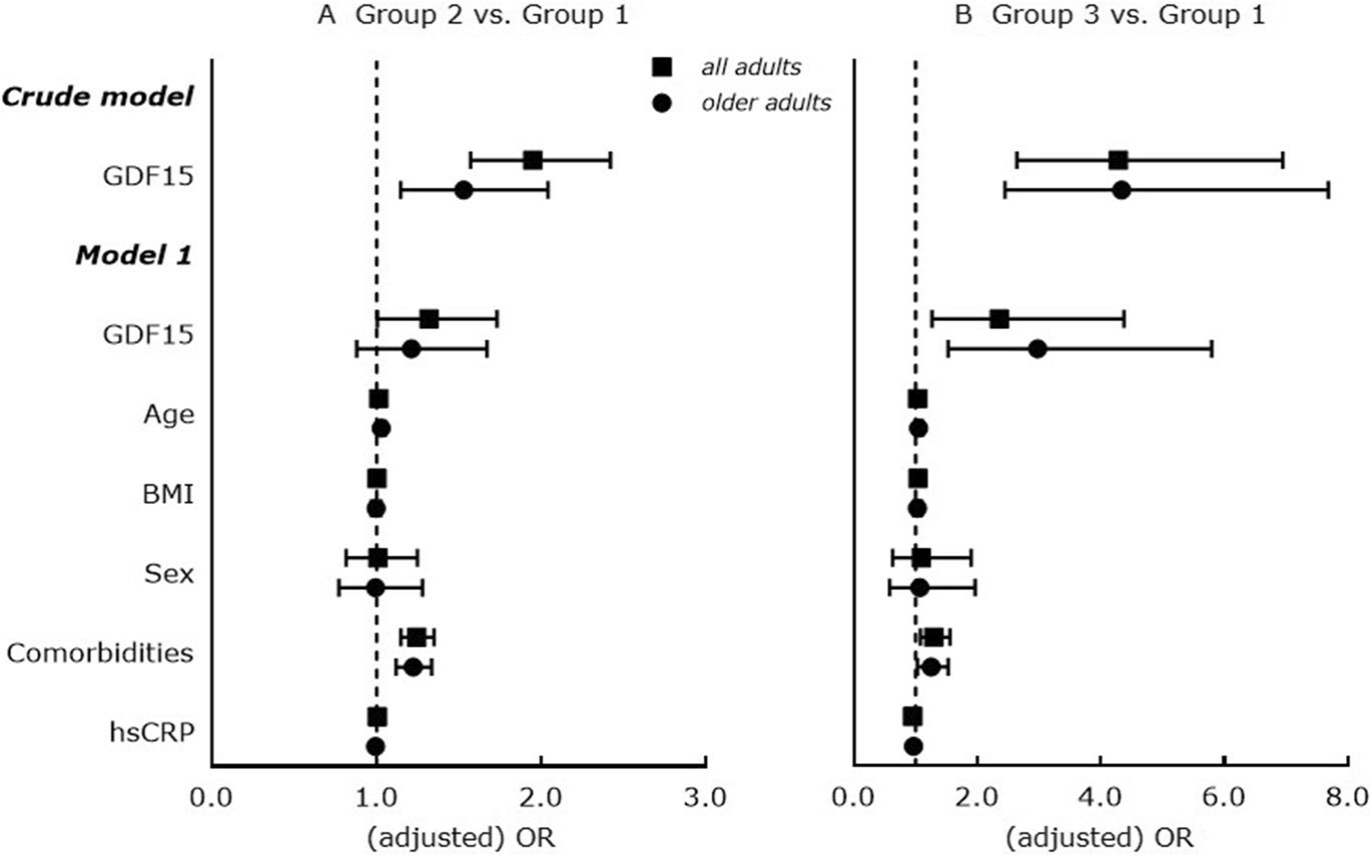
Cross-sectional associations between GDF15 concentrations (considered as per increase in LnGDF15 unit) and cognitive frailty-depression status in all adults (*n* = 2736) and in older adults (≥ 55 years; *n* = 1712) of the MARK-AGE study. Results are displayed as odds ratios (OR) with 95% confidence interval (95% CI); ORs are determined by multinomial logistic regression analysis; vertical line at OR = 1 represents the reference odds ratio. *Crude model*: Cognitive frailty-depression status (Groups 1–3, with Group 1 as reference) as dependent variable and GDF15 concentration (LnGDF15) as covariate. *Model 1*: Cognitive frailty-depression status (Groups 1–3, with Group 1 as reference) as dependent variable and GDF15 concentration (LnGDF15), age [years], BMI [kg/m^2^], sex (men as reference), comorbidities and hsCRP as covariates; all adults: *n* = 2607 (*n* = 129 participants with hsCRP concentration = 0 mg/L were excluded); older adults: *n* = 1666 (*n* = 46 participants with hsCRP concentration = 0 mg/L were excluded). Significance considered at *p* < 0.05. Group 1 = neither-cognitive-frailty-nor-depression, Group 2 = either-cognitive-frailty-or-depression and Group 3 = both-cognitive-frailty-and-depression. BMI, body mass index; GDF15, growth differentiation factor-15; hsCRP, high-sensitive C-reactive protein

**Fig. 2:**
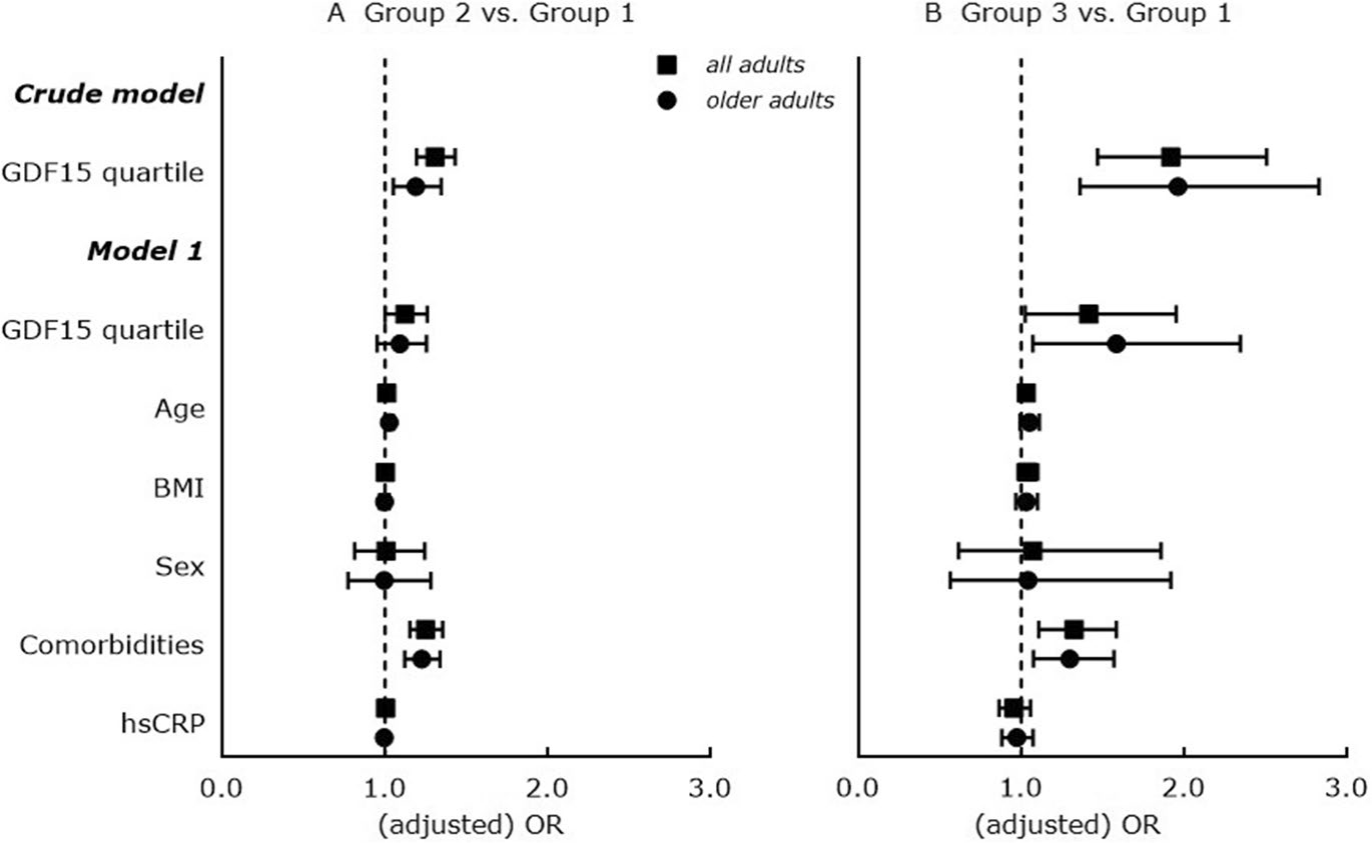
Cross-sectional associations between GDF15 concentrations (considered as per increase in GDF15 quartile) and cognitive frailty-depression status in all adults (*n* = 2736) and in older adults (≥ 55 years; *n* = 1712) of the MARK-AGE study. Results are displayed as odds ratios (OR) with 95% confidence interval (95% CI); ORs are determined by multinomial logistic regression analysis; vertical line at OR = 1 represents the reference odds ratio. *Crude model*: Cognitive frailty-depression status (Groups 1–3, with Group 1 as reference) as dependent variable and GDF15 quartiles (highest quartile as reference) as covariates. *Model 1*: Cognitive frailty-depression status (Groups 1–3, with Group 1 as reference) as dependent variable and GDF15 quartiles (highest quartile as reference), age [years], BMI [kg/m^2^], sex (men as reference), comorbidities and hsCRP as covariates; all adults: *n* = 2607 (*n* = 129 participants with hsCRP concentration = 0 mg/L were excluded); older adults: *n* = 1666 (*n* = 46 participants with hsCRP concentration = 0 mg/L were excluded). Significance considered at *p* < 0.05. Group 1 = neither-cognitive-frailty-nor-depression, Group 2 = either-cognitive-frailty-or-depression and Group 3 = both-cognitive-frailty-and-depression. BMI, body mass index; GDF15, growth differentiation factor-15; hsCRP, high-sensitive C-reactive protein

There are significant positive associations between higher GDF15 concentrations and both-cognitive-frailty-and-depression in all adults (adjusted β = 0.177 [0.044 – 0.310], *p* = 0.009) and in older adults (adjusted β = 0.238 [0.086 – 0.390], *p* = 0.002) (both Table 2**, **model 1). Furthermore, adults with higher GDF15 concentrations show significantly higher odds to have either-cognitive-frailty-or-depression and to have both-cognitive-frailty-and-depression in unadjusted (Fig. 1A and B, crude model, all adults) as well as in age, BMI, sex, comorbidities and hsCRP-adjusted (adjusted OR = 1.319 [1.005 – 1.731], *p* = 0.046; adjusted OR = 2.353 [1.267 – 4.372], *p* = 0.007) (Fig. 1A and B, model 1, all adults) analyses. Similar results are observed regarding significant higher odds to have either-cognitive-frailty-or-depression and to have both-cognitive-frailty-and-depression with increasing GDF15 quartiles in unadjusted (Fig. 2A and B, crude model, all adults) and in age, BMI, sex, comorbidities and hsCRP-adjusted (Fig. 2A and B, model 1, all adults) logistic regression analyses.

**Table 2:** Cross-sectional associations between GDF15 concentrations (considered as change in LnGDF15 unit) and cognitive frailty-depression status in all adults (*n* = 2736) and in older adults (≥ 55 years; *n* = 1712) of the MARK-AGE study

	*LnGDF15* *– all adults*		*LnGDF15* *– older adults*	
*Crude model*	β (95% CI)	*p*-value	β (95% CI)	*p*-value
Group 1 vs. Group 2	0.137 (0.093; 0.181)	< 0.001	0.071 (0.022; 0.119)	0.004
Group 1 vs. Group 3	0.346 (0.228; 0.463)	0.001	0.301 (0.182; 0.419)	< 0.001
*Model 1*				
Group 1 vs. Group 2	0.030 (-0.021; 0.081)	0.247	0.034 (-0.028; 0.096)	0.282
Group 1 vs. Group 3	0.177 (0.044; 0.310)	0.009	0.238 (0.086; 0.390)	0.002
Age [years]	0.019 (0.018; 0.021)	< 0.001	0.021 (0.018; 0.024)	< 0.001
BMI [kg/m^2^]	0.002 (-0.001; 0.006)	0.159	0.005 (0.000; 0.009)	0.029
Sex	0.126 (0.094; 0.158)	< 0.001	0.154 (0.113; 0.195)	< 0.001
Comorbidities [n]	0.052 (0.040; 0.064)	< 0.001	0.051 (0.038; 0.065)	< 0.001
hsCRP [mg/L]	0.016 (0.012; 0.021)	< 0.001	0.014 (0.008; 0.019)	< 0.001

Results are displayed as β coefficient (β) with 95% confidence interval (95% CI); β determined by linear regression analysis. *Crude model*: LnGDF15 as dependent variable and cognitive frailty-depression status (Groups 1–3, with Group 1 as reference) as independent variable. *Model 1*: LnGDF15 as dependent variable and cognitive frailty-depression status (Groups 1–3, with Group 1 as reference), age, BMI, sex (men as reference), comorbidities and hsCRP as independent variables; all adults: *n* = 2607 (*n* = 129 participants with hsCRP concentration = 0 mg/L were excluded); older adults: *n* = 1666 (*n* = 46 participants with hsCRP concentration = 0 mg/L were excluded). Significance considered at *p* < 0.05. Group 1 = neither-cognitive-frailty-nor-depression, Group 2 = either-cognitive-frailty-or-depression and Group 3 = both-cognitive-frailty-and-depression. BMI, body mass index; GDF15, growth differentiation factor-15; hsCRP, high-sensitive C-reactive protein

In older adults, significantly higher odds to have both-cognitive-frailty-and-depression with higher GDF15 concentrations (adjusted OR = 2.973 [1.527 – 5.789], *p* = 0.001; Fig. 1B, model 1, older adults) and with increasing GDF15 quartiles (adjusted OR = 1.585 [1.072 – 2.345], *p* = 0.021; Fig. 2B, model 1, older adults) were confirmed in age, BMI, sex, comorbidities and hsCRP-adjusted analyses.

### High plasma GDF15 concentrations are associated with global cognitive functioning and self-rated depression scores in adults of the MARK-AGE study

In the whole study population, there is both an inverse and a positive significant association of GDF15 concentrations with GCF score (β = -0.463 [-0.691 – -0.234], *p* < 0.001) and SDS score (β = 2.422 [1.633 – 3.211], *p* < 0.001), respectively (Table 3, crude model, all adults). For GCF score, this significant inverse association for GDF15 is lost after confounder adjustments (Table 3, model 1, all adults). For SDS score, there remains a significant positive association of GDF15 in age, BMI, sex, comorbidities and hsCRP-adjusted (adjusted β = 1.413 [0.434 – 2.391], *p* = 0.005) analyses (Table 3, model 1, all adults).

**Table 3:** Cross-sectional associations of both SDS and GCF scores with GDF15 concentrations (LnGDF15) in all adults (*n* = 2736) and in older adults (≥ 55 years; *n* = 1712) of the MARK-AGE study

	*GCF score* *– all adults*		*SDS score* *– all adults*	
*Crude model*	β (95% CI)	*p*-value	β (95% CI)	*p*-value
LnGDF15	-0.463 (-0.691; -0.234)	< 0.001	2.422 (1.633; 3.211)	< 0.001
*Model 1*				
LnGDF15	-0.279 (-0.571; 0.013)	0.061	1.413 (0.434; 2.391)	0.005
Age [years]	-0.010 (-0.022; 0.002)	0.111	-0.003 (-0.044; 0.038)	0.885
BMI [kg/m^2^]	-0.056 (-0.082; -0.030)	< 0.001	-0.015 (-0.102; 0.072)	0.729
Sex	0.664 (0.442; 0.885)	< 0.001	2.256 (1.512; 2.999)	< 0.001
Comorbidities [n]	0.096 (0.005; 0.187)	0.039	1.444 (1.139; 1.749)	< 0.001
hsCRP [mg/L]	0.025 (-0.009; 0.060)	0.151	-0.014 (-0.130; 0.101)	0.806
	*GCF score* *– older adults*		*SDS score* *– older adults*	
*Crude model*	β (95% CI)	*p*-value	β (95% CI)	*p*-value
LnGDF15	-0.402 (-0.747; -0.057)	0.022	1.534 (0.423; 2.646)	0.007
*Model 1*				
LnGDF15	-0.223 (-0.611; 0.165)	0.259	1.217 (0.003; 2.430)	0.049
Age [years]	-0.019 (-0.046; 0.009)	0.180	0.011 (-0.076; 0.097)	0.811
BMI [kg/m^2^]	-0.070 (-0.104; -0.036)	< 0.001	-0.047 (-0.154; 0.059)	0.386
Sex	0.663 (0.364; 0.963)	< 0.001	2.278 (1.340; 3.215)	< 0.001
Comorbidities [n]	0.111 (0.002; 0.221)	0.047	1.301 (0.957; 1.645)	< 0.001
hsCRP [mg/L] ^1^	0.030 (-0.016; 0.076)	0.199	0.008 (-0.136; 0.152)	0.917

Results are displayed as β coefficient (β) with 95% confidence interval (95% CI); β determined by linear regression analysis. *Crude model*: GCF score or SDS score as dependent variable and LnGDF15 as independent variable. *Model 1:* GCF score or SDS score as dependent variable and LnGDF15, age, BMI, sex (men as reference), comorbidities and hsCRP as independent variables; all adults: *n* = 2607 (*n* = 129 participants with hsCRP concentration = 0 mg/L were excluded); older adults: *n* = 1666 (*n* = 46 participants with hsCRP concentration = 0 mg/L were excluded). Significance considered at *p* < 0.05. BMI, body mass index; GCF, global cognitive functioning; GDF15, growth differentiation factor-15; hsCRP, high-sensitive C-reactive protein; SDS, self-rating depression scale

In older adults, similar results are found showing significant associations of GDF15 concentrations with GCF scores only in unadjusted analyses, and with SDS scores in unadjusted as well as confounder-adjusted (adjusted β = 1.217 [0.003 – 2.430)], *p* = 0.049) analyses (Table 3, older adults). Our results indicate that the depression-defining SDS score might independently be associated with GDF15, whereas the cognitive frailty-defining GCF score might not be independently related with GDF15.

### High plasma GDF15 concentrations are associated with cognitive frailty and depression separately in adults of the MARK-AGE study

**Fig. 3:**
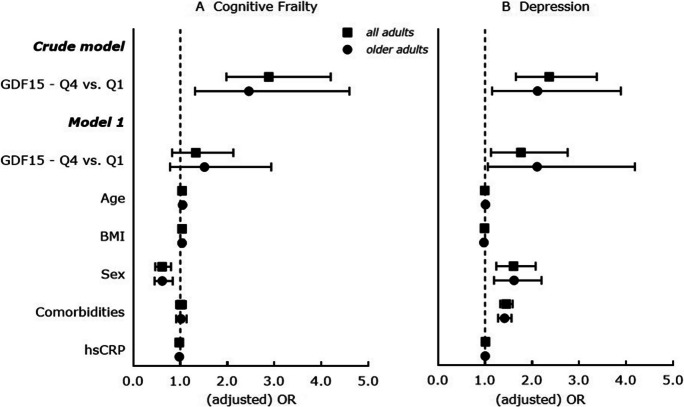
Cross-sectional associations between GDF15 (considered as per increase in GDF15 quartile) and **A**. cognitive frailty and **B**. depression status in all adults (*n* = 2736) and in older adults (≥ 55 years; *n* = 1712) of the MARK-AGE study. Results are displayed as odds ratios (OR) with 95% confidence interval (95% CI); ORs are determined by multinomial logistic regression analysis; vertical line at OR = 1 represents the reference odds ratio. *Crude model*: Cognitive frailty status (cognitive frail vs. non-frail) or depression status (depressed vs. non-depressed) as dependent variable and GDF15 quartiles (lowest quartile (Q1) as reference) as independent variable. *Model 1*: Cognitive frailty status (cognitive frail vs. non-frail) or depression status (depressed vs. non-depressed) as dependent variable and GDF15 quartiles (lowest quartile (Q1) as reference), age [years], BMI [kg/m^2^], sex (men as reference), comorbidities and hsCRP as independent variables; all adults: *n* = 2607 (*n* = 129 participants with hsCRP concentration = 0 mg/L were excluded); older adults: *n* = 1666 (*n* = 46 participants with hsCRP concentration = 0 mg/L were excluded). Significance considered at *p* < 0.05. BMI, body mass index; GDF15, growth differentiation factor-15; hsCRP, high-sensitive C-reactive protein

In a next step, cognitive frailty and depression were analyzed separately. Here, adults with higher GDF15 concentrations have significantly higher odds to be cognitively frail and to have depressive symptoms in unadjusted and in age, BMI, sex, comorbidities and hsCRP-adjusted analyses (Supplemental Table S3**,** all adults). Furthermore, the likelihood to be cognitively frail is significantly higher with increasing GDF15 quartiles, whereas this association is not confirmed in confounder adjusted models (Fig. 3A, all adults). The likelihood to have depressive symptoms is significantly increased with higher GDF15 quartiles in unadjusted as well as adjusted analyses (highest quartile vs. lowest quartile: adjusted OR = 1.763 [1.127 – 2.758], *p* = 0.013; Fig. 3B, all adults).

In older adults, there are significantly higher odds to be cognitively frail in unadjusted and confounder adjusted analyses (adjusted OR = 1.566 [1.065 – 2.302], *p* = 0.022) (Supplemental Table S3, older adults). Moreover, the likelihood to be cognitively frail is significantly higher with higher GDF15 quartiles only in unadjusted models and not after confounder adjustments (Fig. 3A, older adults). The likelihood to have depressive symptoms is also significantly increased with higher GDF15 quartiles in unadjusted as well as adjusted analyses (highest quartile vs. lowest quartile: adjusted OR = 2.107 [1.060 – 4.189], *p* = 0.034; Fig. 3B, older adults). Our results indicate that circulating GDF15 might age, BMI, sex, comorbidities and hsCRP-independently associated with cognitive frailty and depressive symptoms.

## Discussion

Cognitive frailty and depression may coexist and pathophysiological mechanisms overlap, but studies evaluating the relationship of circulating GDF15 with combined cognitive frailty and depression are lacking. This is the first study revealing that GDF15 concentrations are significantly altered in adults with having both-cognitive-frailty-and-depression and that high plasma GDF15 concentrations are significantly associated with a higher occurrence and likelihood of both-cognitive-frailty-and-depression in adults independently of age, BMI, sex, comorbidities and hsCRP. Importantly, these significant higher GDF15 concentrations and significant associations of high GDF15 with a higher likelihood for both-cognitive-frailty-and-depression are confirmed in older adults (Figs. 1 and 2, and Supplemental Table S1). Furthermore, we showed that high plasma GDF15 is significantly associated with cognitive frailty and depression separately, where the associations with both conditions might be independent of age, BMI, sex, comorbidities and hsCRP. This is true for adults of the whole study population and for older adults only (Fig. 3 and Supplemental Table S3). Moreover, high GDF15 is significantly age, BMI, sex, comorbidities and hsCRP-independently related only to self-rated depression scores but not to GCF scores in adults of the whole study population and in older adults (Table 3).

Since GDF15 can act as a pro-inflammatory stress signal mediating inflammatory response, the potential detrimental effects of (prolonged) elevated GDF15 concentrations might be related to “inflammaging”. Inflammaging represents a chronic low-grade inflammation in higher age and is associated with age-related diseases [31, 32]. In accordance to this, there was a significant positive association between GDF15 and hsCRP in our study (ρ = 0.206, *p* < 0.001; Supplemental Table S2). Moreover, hsCRP was significantly positively associated with GDF15 in all adults (adjusted β = 0.016 [0.012; 0.021], *p* < 0.001) and in older adults (adjusted β = 0.014 [0.008; 0.019], *p* < 0.001) (both Table 2, model 1) of the MARK-AGE cohort, subsequently adding to the relation between GDF15 and inflammation. Additionally, GDF15 is also part of the SASP, which is associated with less resilience of cells (e.g. neurons) against external (i.e. lifestyle) and internal (i.e. biological) stressors.

GDF15 is considered as a biomarker of aging [1, 3], and accordingly we found a significant relation between age and GDF15 in our study. Since we also observed an age-dependent increase in the prevalence of cognitive frailty as well as depression, we focused our analyses on older adults. Interestingly, age was still associated with both-cognitive-frailty-and-depression as well as GDF15 in our older participants, but not with global cognitive functioning and self-rated depression scores in adjusted regression models. Beside age, BMI might also affect GDF15 concentrations, since GDF15 is involved in weight as well as appetite regulation [33, 34]. In our study, BMI is similar between the three cognitive frailty-depression groups, but is associated with GDF15 concentrations in adjusted regression analyses. Sex distribution was also similar between the three cognitive frailty-depression groups without a significant association with both-cognitive-frailty-and-depression; however, sex was significantly associated with GFC and SDS scores as well as with GDF15 within the adjusted regression models. Sex-specific analyses in our study showed that men had significantly higher GFD15 concentrations as well as lower GCF scores compared to women, that women had significantly higher SDS scores than men, and that both sexes were similar in age (data not shown). This is in accordance with the previous finding that older male patients had higher GDF15 levels than female patients [35]. Although underlying mechanisms are not clear so far, this might be explained by sex hormone effects on GDF15 [36].

GDF15 has been linked previously to age- and cognition-related conditions. In older subjects, high systemic GDF15 was associated with lower global cognition, worse cognitive performance and cognitive impairment [7, 37, 38]. Since high GDF15 levels were linked to brain structural degenerations in older adults [37, 38], changes in brain structure possibly link high GDF15 concentrations to deteriorating cognitive functioning [8]. GDF15 is expressed in the human brain, probably predominantly by neurons, and its expression correlates positively with IL-6 expression [39]. In vitro modulation of GDF15 expression affects mitochondrial gene expression and morphology, and inflammatory marker suggesting an inflammatory response to mitochondrial dysfunction in which GDF15 is likely part of a network aimed at modulating this response [39]. Increased GDF15 was also associated with pro-inflammatory markers and a significantly higher risk for post-stroke depression [40]. Although not all studies have implicated GDF15 as an independent inflammatory biomarker for late-life depression [41], late-life depression was linked to high GDF15 levels, which were further related to lower cognitive functioning in adults with depression [42]. Here it has been suggested that GDF15 can be a biological pathway between depression and cognitive aging [42].

Our findings are subject to limitations. The cross-sectional design of our analyses does not allow to draw conclusions on causality and whether there is a direct link or involvement of GDF15 with disease development. Prospective longitudinal studies are needed in the future to address such an involvement. Moreover, including further confounders for GDF15 or for both conditions might improve our analyses. Data on circulating sex hormones might also improve our results, and sex-specific analyses should be considered in future studies. However, we adjusted all analyses for age, BMI and sex as well as for having comorbidities (reflecting health status) and for hsCRP concentrations (reflecting inflammation), which are known confounding and associated factors of GDF15, ultimately strengthening our results. Combined cognitive-frailty-and-depression was not frequent within the whole study population, although there was an increasing prevalence with higher age-groups (up to 10.3% within the age-group 75 + years). Our study population also consists of participants from different European countries, therefore reflecting a broad geographical distribution and a variety of lifestyles.

In conclusion, high plasma GDF15 concentrations are significantly associated with combined cognitive-frailty-and-depression status, with both conditions separately as well as with global cognitive functioning and self-rated depression scores in old as well as young community-dwelling adults of the MARK-AGE study. Further studies need to evaluate the exact role of GFD15 in pathophysiological mechanisms of both conditions.

### Supplementary Information

Below is the link to the electronic supplementary material.

Supplementary file1 (JPG 83 KB)

Supplementary file2 (DOCX 35 KB)
