# Supplementary figures and images for: Associations of circulating GDF15 with combined cognitive frailty and depression in older adults of the MARK-AGE study
Source: GeroScience. 2023 Sep 16;46(2):1657–69. doi: 10.1007/s11357-023-00902-6 (PMC10828354; doi:10.1007/s11357-023-00902-6)

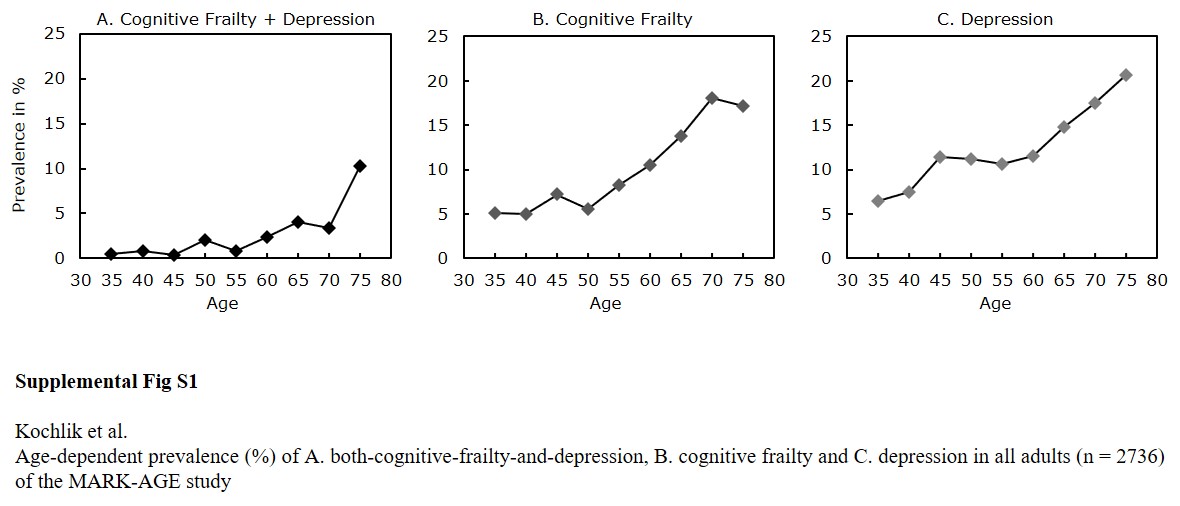

Supplement: Supplementary file 1 — Supplementary file1 (JPG 83 KB) [file 11357_2023_902_MOESM1_ESM.jpg]
