# Supplementary material for: Associations of circulating GDF15 with combined cognitive frailty and depression in older adults of the MARK-AGE study
Source: GeroScience. 2023 Sep 16;46(2):1657–69. doi: 10.1007/s11357-023-00902-6 (PMC10828354; doi:10.1007/s11357-023-00902-6)
Supplement: Supplementary file 2 — Supplementary file2 (DOCX 35 KB) [file 11357_2023_902_MOESM2_ESM.docx]

**Supplemental Table S1 Participant characteristics and GDF15 concentrations according to cognitive frailty and depression status of older adults (≥ 55 years; n=1712)** **of the MARK-AGE study**

|  | no-cognitive-frailty-no-depression | either-cognitive-frailty-or-depression | both-cognitive-frailty-and-depression | p-value |
| --- | --- | --- | --- | --- |
| N [%] | 1314 (76.8) | 350 (20.4) | 48 (2.8) |  |
| GCF score [points] | 0.542 ± 2.501 ^a^ | -1.354 ± 3.872 ^b^ | -4.575 ± 1.084 ^c^ | **< 0.001** |
| SDS score [points] | 35.7 ± 7.2 ^a^ | 47.6 ± 10.2 ^b^ | 56.9 ± 6.3 ^c^ | **< 0.001** |
| Women [n (%)] | 672 (76.3) | 185 (21.0) | 24 (2.7) | 0.832^#^ |
| Men [n (%)] | 642 (77.3) | 165 (19.9) | 24 (2.9) |  |
| Age [years] | 64.6 ± 5.5 ^a^ | 65.9 ± 5.8 ^b^ | 67.4 ± 5.3 ^b^ | **0.002** |
| BMI [kg/m^2^] | 26.2 (5.3) | 26.5 (5.2) | 27.2 (6.3) | 0.117 |
| Comorbidities [n] | 1.0 (2.0) | 2.0 (2.0) | 2.0 (2.0) | **< 0.001** |
| hsCRP [mg/L] ^1^ | 1.42 (2.13) | 1.36 (1.99) | 1.18 (1.58) | 0.742 |
| GDF15 [pg/mL] ^2^ | 894.1  (874.5; 914.2) ^a^ | 959.6  (919.1; 1002.0) ^b^ | 1207.7  (1071.5; 1361.2) ^c^ | **< 0.001** |
|  | 903.3  (884.; 922.9) ^a^ | 940.1  (903.8; 977.9) ^a^ | 1128.9  (1010.1; 1261.6) ^b^ | **< 0.001*** |

Data are shown as mean ± standard deviation or as median (interquartile range). ^1^ hsCRP: n = 1666 (n = 46 participants with hsCRP concentration = 0 mg/L were excluded). ^2^ Data for GDF15 concentrations are shown as geometric mean (95% confidence interval (95% CI)) of back-transformed LnGDF15 values. Differences between groups are determined by one-way ANOVA with Bonferroni post-hoc test or Kruskal-Wallis-test for continuous variables and by ^#^ Chi-square-test for categorical variables. * ANCOVA: adjusted for age, BMI and sex. Superscript letters indicate statistically significant differences between frailty groups. Significance considered at p < 0.05. BMI, body mass index; GCF, global cognitive functioning; GDF15, growth differentiation factor-15; hsCRP, high-sensitive C-reactive protein; SDS, self-rating depression scale.

**Supplemental Table S2 Correlations between SDS scores, GCF scores, GDF15 concentrations, age, BMI** **and hsCRP in all adults** **(n = 2736) of the MARK-AGE study**

|  | GDF15 [pg/mL] | Age [years] | SDS score | GCF score |
| --- | --- | --- | --- | --- |
| Age [years] | ρ = 0.601^**^ | / | / | / |
| SDS score | ρ = 0.105^**^ | r = 0.112^**^ | / | / |
| GCF score | ρ = -0.106^**^ | ρ = -0.099^**^ | ρ = -0.022 | / |
| BMI [kg/m^2^] | ρ = 0.195^**^ | ρ = 0.193^**^ | ρ = 0.023 | ρ = -0.142^**^ |
| hsCRP [mg/L] ^1^ | ρ = 0.206^**^ | ρ = 0.122^**^ | ρ = 0.018 | ρ = -0.021 |

Spearman rank correlation coefficient (ρ); Pearson correlation coefficient (r). ^1^ hsCRP: n = 2607 (n = 129 participants with hsCRP concentration = 0 mg/L were excluded). ** p < 0.01. BMI, body mass index; GCF, global cognitive functioning; GDF15, growth differentiation factor-15; hsCRP, high-sensitive C-reactive protein; SDS, self-rating depression scale.

**Supplemental Table S3. Cross-sectional associations between GDF15 (considered as per increase in LnGDF15 unit) and both depression and cognitive frailty status in all adults (n = 2736) and in older adults (≥ 55 years; n = 1712) of the MARK-AGE study**

|  | *Cognitive Frailty status*  *– all adults* | | *Depression status*  *– all adults* | |
| --- | --- | --- | --- | --- |
| ***Crude model*** | **OR (95% CI)** | **p-value** | **OR (95% CI)** | **p-value** |
| **LnGDF15** | 2.412 (1.858; 3.131) | < 0.001 | 1.907 (1.494; 2.436) | < 0.001 |
| ***Model 1*** |  |  |  |  |
| **LnGDF15** | 1.499 (1.067; 2.107) | 0.020 | 1.415 (1.039; 1.928) | 0.028 |
| **Age [years]** | 1.034 (1.018; 1.051) | 0.000 | 0.998 (0.983; 1.012) | 0.732 |
| **BMI [kg/m^2^]** | 1.036 (1.005; 1.067) | 0.021 | 0.988 (0.960; 1.016) | 0.392 |
| **Sex** | 0.620 (0.473; 0.812) | 0.001 | 1.615 (1.247; 2.092) | < 0.001 |
| **Comorbidities [n]** | 1.000 (0.902; 1.109) | 0.998 | 1.438 (1.312; 1.5769 | < 0.001 |
| **hsCRP [mg/L]** | 0.977 (0.934; 1.021) | 0.297 | 1.008 (0.972; 1.045) | 0.680 |
|  | ***Cognitive Frailty status***  ***– older adults*** | | ***Depression status***  ***– older adults*** | |
| ***Crude model*** | **OR (95% CI)** | **p-value** | **OR (95% CI)** | **p-value** |
| **LnGDF15** | 2.107 (1.514; 2.934) | < 0.001 | 1.684 (1.217; 2.329) | 0.002 |
| ***Model 1*** |  |  |  |  |
| **LnGDF15** | 1.566 (1.065; 2.302) | 0.022 | 1.420 (0.984; 2.050) | 0.061 |
| **Age [years]** | 1.047 (1.018; 1.077) | 0.001 | 1.011 (0.984; 1.039) | 0.443 |
| **BMI [kg/m^2^]** | 1.036 (1.002; 1.072) | 0.038 | 0.976 (0.944; 1.009) | 0.156 |
| **Sex** | 0.628 (0.461; 0.854) | 0.003 | 1.632 (1.199; 2.221) | 0.002 |
| **Comorbidities [n]** | 1.001 (0.896; 1.117) | 0.992 | 1.400 (1.265; 1.549) | < 0.001 |
| **hsCRP [mg/L]** | 0.976 (0.928; 1.026) | 0.340 | 1.000 (0.956; 1.045) | 0.992 |

Results are displayed as odds ratios (OR) with 95% confidence interval (95% CI); ORs are determined by logistic regression analysis.

***Crude model***: Cognitive frailty status (cognitive frail vs. non-frail) or depression status (depressed vs. non-depressed) as dependent variable and LnGDF15 as independent variable.

***Model 1***: Cognitive frailty status (cognitive frail vs. non-frail) or depression status (depressed vs. non-depressed) as dependent variable and LnGDF15, age, BMI, sex (men as reference), comorbidities and hsCRP as independent variables; all adults: n = 2607 (n = 129 participants with hsCRP concentration = 0 mg/L were excluded); older adults: n = 1666 (n = 46 participants with hsCRP concentration = 0 mg/L were excluded).

Significance considered at p < 0.05. BMI, body mass index; GDF15, growth differentiation factor-15; hsCRP, high-sensitive C-reactive protein.
